# Accuracy of Pelvic Ultrasound in Diagnosing Adnexal Torsion

**DOI:** 10.1155/2019/1406291

**Published:** 2019-07-01

**Authors:** Labib Ghulmiyyah, Anwar Nassar, Dib Sassine, Sally Khoury, Joseph Nassif, Hadi Ramadan, Elie Najem, Ghina Berjawi

**Affiliations:** ^1^Department of Obstetrics and Gynecology, American University of Beirut Medical Center, Lebanon; ^2^Department of Diagnostic Radiology, American University of Beirut Medical Center, Lebanon

## Abstract

Ovarian/adnexal torsion is a rather frequent occurrence in women of reproductive age group worldwide. Etiologies are quite diverse with ovarian lesions and corpus luteal cysts being the most two common. Pelvic or intravaginal ultrasound remains the first-line imaging modality used for diagnosis and evaluation of suspected ovarian/adnexal torsion. In this study, we have adopted a case-based statistical analysis to identify important sonographic markers and further evaluated their contribution in identifying ovarian torsion. Our study successfully determined the important sonographic markers. Our observation and analysis suggest that ovarian enlargement is the most sensitive marker. Ovarian edema was found to be the most specific marker to identify the ovarian torsion with higher level of accuracy and confidence. This pioneer study will provide valuable information and direction to the medical practitioners and radiologists for better diagnosis. Further studies with large sample size will help in establishing our findings universally.

## 1. Introduction

Adnexal torsion accounts for almost 3% of surgical emergencies [1]. It involves twisting of the ovary and/or fallopian tube leading to either a necrotic ovary or an undamaged ovary with impaired vascularization. This incidence is most common in women aged between 14 and 45 years. To prevent the loss of the ovary or adnexa and severe conditions such as thrombophlebitis or peritonitis, delay in the diagnosis should be avoided [2]. The presence of an ovarian mass of at least 5 cm is the primary risk factor that can cause torsion [3, 4].

Diagnosis of an ovarian torsion is challenging due to lack of sensitivity and specificity of its clinical signs [5]. Mostly, patients with ovarian torsion show abdominal pain as the major clinical symptom [6, 7]. To the present, the diagnosis of ovarian torsion has been based on clinical findings.

Reports from existing evidence suggest that sonography along with ovarian vasculature Doppler flow studies help in the correct diagnosis of only 66% of surgical cases [1]. The ultrasonography based diagnostic technique is known to be operator-dependent and, thus, may involve human error.

Our study aims to identify the sonographic marker(s) that are most helpful in making an accurate diagnosis of adnexal torsion. We also aim to assess the accuracy and predictive value of such identified sonographic markers to aid the early intervention.

## 2. Materials and Methods

This study was approved by the Institutional Review Board at the American University of Beirut. The study subjects were retrieved from the Department of Diagnostic Radiology of the American University of Beirut Medical Center (AUBMC) database. All women aged between 14 and 45 years presenting to AUBMC Emergency Department between July 2009 and February 2015 with acute lower abdominal pain suggestive of ovarian/adnexal were identified. Among the selected subjects, only those with positive ultrasound findings suggestive of ovarian/adnexal torsion and underwent laparoscopic surgery were included in this study.

The ultrasound machine used for the acquisition of sonographic images is a PHILIPS EPIQ 5G. A curvilinear probe C5-1 was used with a 5 MHz frequency. Both axial and sagittal cuts were obtained.

Relevant information including patient demographics, past medical and surgical history, and operative and pathology notes were collected from the electronic medical records. All retrieved data were then tabulated into an excel sheet. To avoid bias, a second sheet containing only the patients' study number, demographics, and clinical history was sent to a specialized women imaging Diagnostic Radiologist at the Diagnostic Radiology department at AUBMC for reevaluation of the ultrasound images. Ultrasound findings and the sonographic markers—ovarian edema, ovarian enlargement, ovarian location, and presence/absence of an ovarian cyst/mass, ovarian blood flow, fluid in the pouch of Douglas, fluid around the ovary, distention of fallopian tubes, and bleeding within ovaries— were then added to the excel sheet and sent for statistical analysis.

Initial data tabulation was done in a Microsoft Excel sheet and later loaded into the Statistical Package for Social Sciences (SPSS) package version 22 for analyses. Descriptive statistical analysis was carried out by reporting the number and percentage of the categorical variables whereas continuous variables were summarized by mean and standard deviation. Fisher's Exact test was used to compare the presence of sonographic signs between the samples with and without laparoscopic evidence of torsion. To assess the statistical outcome, the sensitivity, specificity, positive predictive value (PPV), and negative predictive value (NPV) were calculated. During these analyses, 95% confidence intervals (CI) were considered for each statistical measure.

## 3. Results

Table 1 summarizes the characteristics of the 37 patients who met the inclusion criteria. The baseline characteristics were found to be similar in the torsion and no torsion groups (Table 2).

Table 3 represents the frequency of various sonographic markers associated with ovarian torsion in women with and without evidence of ovarian torsion determined by laparoscopy. In patients with ovarian torsion, the most frequent sonographic markers observed were relative enlargement of the affected ovary, ovarian edema, and the presence of an ovarian mass/cyst. Ovarian edema and ovarian enlargement were both found to be statistically significant markers (p = 0.03).

Three out of 10 ovarian torsion cases had abnormal ovarian location noted on ultrasound (p = 0.02).

Interestingly, none of the 10 torsion cases had abnormal ovarian blood flow using Doppler ultrasound.

Table 4 summarizes the sensitivity, specificity, PPV, and NPV of the various sonographic markers. Ovarian enlargement was found to be the most sensitive marker (80%) with a specificity of 63%. On the other hand, ovarian edema was found to be the most specific marker (100%) and a CI of 0.8-1. In this study, abnormal ovarian blood flow was not found to be sensitive as the calculated sensitivity was 0%.

Table 5 shows the frequency of the combination of sonographic markers found during the scan in patients suspected to have ovarian torsion. It was found that 11 had only 1 marker, whereas 15 had 3 or more markers.

It was observed that when only a single marker was detected on ultrasound, the sensitivity approached 90% whereas the presence of 3 combined markers increased the specificity to 96% (Table 6). Therefore, we calculated the Youden score which simultaneously reflects the sensitivity and the specificity for a combination of markers. It was observed that a combination of 2 markers detected on ultrasound had the highest Youden score of 0.4.

## 4. Discussion

At present, there is no reliable method to confirm the diagnosis of adnexal torsion preoperatively. To prevent the loss of the ovarian function and its potential association with fertility problems, an early diagnosis of an adnexal torsion is needed [8]. Moreover, delay in diagnosis and treatment or mistake in diagnosis may lead to potentially fatal thrombophlebitis or peritonitis [8, 9].

This study demonstrates the importance of combining both clinical and ultrasonographic findings together to diagnose ovarian torsion with better accuracy and reliability.

The existing literature suggests that torsion occurs in the right side in most of the cases accounting for almost 67-71% [10, 11]. This is since the right uteroovarian ligament is physiologically longer than its left counterpart. The presence of the sigmoid colon on the left side reduces the space needed and may aid in the occurrence of torsion. Our study showed that 60% of the adnexal torsion occurred in the left side.

Our findings will help in future diagnosis and early detection of ovarian torsion and will guide the medical practitioners or radiologists in handling such cases.

We observed that ovarian enlargement was the most sensitive marker in our series in diagnosing ovarian torsion. The sensitivity found was 80% and specificity of 63% was achieved for this specific marker. Similar outcome was also shown in another case series of 63 ovarian torsion cases by Mashiach et al., where ovarian enlargement was the most sensitive sonographic finding with a sensitivity of 85% and specificity of 18.8% [1].

Comparison of the enlarged ovarian size with the unaffected ovarian size in normal subjects is of major importance. However, normal looking ovaries do not necessarily rule out the possibility of ovarian torsion [12].

According to Pena et al. [10], 60% of the cases of torsion are missed by Doppler, while its positive predictive value was 100.

Abnormal flow detected by Doppler sonography can be highly predictive of adnexal torsion and is therefore useful in the diagnosis of ovarian torsion. In fact, several studies have previously concluded that completely normal venous waveforms are very unlikely in cases of ovarian torsion [13, 14]. However, when normal flow is detected, it does not necessarily exclude an ovarian torsion; in fact, torsion can be missed in 60% of cases [10]. In addition, Murat et al. (xxxx) also reported velocity loss in Doppler sonography in 46.4% of the patients.

Unlike the literature, in our case series, no abnormal arterial or venous Doppler flow was noted in any of the 10 ovarian torsion cases, not enabling us to calculate the positive predictive value (PPV) in this case.

Therefore, relying on normal Doppler sonography could have resulted in missing all cases of torsion. Normal Doppler studies should not delay surgical intervention in the presence of a high clinical index of suspicion.

Our study suggests that using multiple sonographic markers can be helpful to confirm the presence of torsion and initiate surgical intervention in a timely fashion. Marker combination was able to increase the specificity value of the test. Using two sonographic markers increased the specificity up to 70% compared with 44% specificity with the use of a single marker.

Interestingly, incorporating more than two of such markers would eventually reduce the overall sensitivity. Our results also showed that a single torsion marker achieved a sensitivity of 90% whereas considering three markers reduced the sensitivity up to 40%. Similar findings were observed by Reuven et al., where the combination of sonographic markers displayed higher specificity and lower sensitivity values [1].

For better evaluation and optimal outcome and to understand the impact of the combination of the markers with best possible specificity and sensitivity, we considered Youden score.

The highest observed Youden score was 0.4, suggesting that combination of any two sonographic markers related to the ovarian torsion could provide the best possible sensitivity and specificity for the accounted dataset.

Our study has several limitations the most important of which is the small sample size. Despite this, we believe that our study is a pioneer study in Lebanon and in the Middle East region in this direction. Utmost care was taken to extract the best possible results through extensive and reliable statistical measures such as Youden scoring and blinded evaluation of the sonographic images by the radiologist.

With time, along with the increment of complications such as PCOD and other, the number of cases of ovarian torsion is increasing. Reports are available from all geographical regions in this context. Ultrasonography is the most commonly used and cost-effective method of diagnosis so far. Early and accurate diagnosis remains a challenge for ovarian torsion. Early detection of ovarian torsion also helps in the further treatment decision which may have less complication. Reports suggest that MRI and CT scan have been attempted as an alternative protocol towards better diagnosis [15–17]. But ultrasound remains the most cost-effective, easily accessible diagnostic measure for adnexal torsion all over the world.

Studies have reported that ultrasound-based methods are quite reliable in diagnosing torsions where significant signs such as abnormal blood flow [18], positive whirlpool sign [19], and other markers could serve as best predictors and confirm the presence of adnexal torsion. Therefore, our study will help in considering specific markers which could be the best possible markers in terms of their sensitivity and specificity while doing such diagnosis.

## 5. Conclusion

The increment in the number of cases of adnexal torsion demands specific, quick, and accurate diagnostic measure. Ultrasound has been the most successful technique in this scenario. Yet, considering the consequences and probable loss of the patient due to delayed diagnosis or misdiagnosis, better detection accuracy is required.

The common symptoms are unspecific in nature in most of the cases of adnexal torsion and sometimes Doppler method may not be able to detect the abnormalities as well. At present, the accurate diagnosis of ovarian torsion can only be achieved and treated by surgery. Therefore, increased sensitivity and specificity in diagnosis through ultrasound is an urgent need.

Along with the clinical suspicion and estimations, this study will provide ample valuable information towards understanding the impact of the sonographic markers during the diagnosis of ovarian torsion accurately.

## Figures and Tables

**Table 1:** Patient characteristics.

	N=37
Age*∗*	27.16 ± 6.58
Gravidity*∗*	1.05 ± 1.54
Parity*∗*	0.84 ± 1.32
Smoking*∗∗*	
No	36 (97.3)
Yes	1 (2.7)
Past Surgical History*∗∗*	
None	17 (45.9)
Abdomino-Pelvic	12 (32.4)
Other	8 (21.6)
Body Mass Index*∗*	24.01 ± 4.10
History of PCOS*∗∗*	
Yes	3 (8.1)
No	34 (91.9)
Pain location*∗∗*	
Left	13 (35.1)
Right	20 (54.1)
Mid	4 (10.8)

**∗**Mean ± SD; *∗∗*n (%); PCOS=polycystic ovary syndrome.

**Table 2:** Patient characteristics in the two patient groups.

Variables	Negative^†^ (N=27)	Positive^††^ (N=10)	P-Value
Age*∗*	27.15 ± 6.91	27.20 ± 5.94	1
Gravidity*∗*	1.11 ± 1.58	0.90 ± 1.52	0.67
Parity*∗*	0.89 ± 1.34	0.70 ± 1.34	0.72
Smoking*∗∗*			1
No	26 (96.3)	10 (100.0)	
Yes	1 (3.7)	0 (0.0)	
Past Surgical History*∗∗*			0.51
None	11 (40.7)	6 (60.0)	
Abdomino-Pelvic	9 (33.3)	3 (30.0)	
Other	7 (25.9)	1 (10.0)	
Body Mass Index*∗*	23.95 ± 4.31	24.16 ± 3.66	0.65
History of PCOS*∗∗*			0.55
Yes	3 (11.1)	0 (0.0)	
No	24 (88.9)	10 (100.0)	
Pain location*∗∗*			0.16
Left	7 (25.9)	6 (60.0)	
Right	16 (59.3)	4 (40.0)	
Mid	4 (14.8)	0 (0.0)	

Negative^†^ refers to patients with no proven ovarian torsion determined by laparoscopy.

Positive^††^ refers to patients with proven ovarian torsion determined by laparoscopy.

**∗**Mean ± SD; *∗∗*n (%); PCOS=polycystic ovary syndrome.

**Table 3:** Presence of the various sonographic markers in the torsion and no torsion groups.

Variables	Negative^†^ (N=27)	Positive^††^ (N=10)	P-Value
Ovarian edema	0 (0.0)	4 (40.0)	0.003
Ovarian enlargement	10 (37.0)	8 (80.0)	0.03
Ovarian cyst or mass	11 (40.7)	6 (60.0)	0.3
Abnormal ovarian location	0 (0.0)	3 (30.0)	0.002
Abnormal ovarian blood flow	0 (0.0)	0 (0.0)	NA
Free fluid in pouch of Douglas	6 (22.2)	2 (20.0)	1
Fluid around the ovary	0 (0.0)	0 (0.0)	NA
Distended fallopian tube	0 (0.0)	0 (0.0)	NA
Sites of bleeding within the affected ovary	0 (0.0)	0 (0.0)	NA

Negative^†^ refers to patients with no proven ovarian torsion determined by laparoscopy.

Positive^††^ refers to patients with proven ovarian torsion determined by laparoscopy.

Data presented as n (%).

**Table 4:** The observed outcome of sensitivity, specificity, PPV, and NPV.

Signs	Sensitivity	Specificity	PPV	NPV
	(95% CI)	(95% CI)	(95% CI)	(95% CI)
Ovarian edema	0.40 (0.14 - 0.73)	1.00 (0.84 - 1.00)	1.00 (0.40 - 1.00)	0.82 (0.64 - 0.92)
Ovarian enlargement	0.80 (0.44 - 0.96)	0.63 (0.42 - 0.80)	0.44 (0.22 - 0.69)	0.89 (0.65 - 0.98)
Ovarian cyst or mass	0.60 (0.27 - 0.86)	0.59 (0.39 - 0.77)	0.35 (0.15 - 0.61)	0.80 (0.56 - 0.93)
Abnormal ovarian location	0.30 (0.08 - 0.65)	1.00 (0.84 - 1.00)	1.00 (0.40 - 1.00)	0.79 (0.61 - 0.91)
Abnormal ovarian blood flow	0.00 (0.00 - 0.34)	1.00 (0.84 - 1.00)	NA	0.73 (0.56 - 0.86)
Free fluid in the pouch of Douglas	0.20 (0.03 - 0.56)	0.78 (0.57 - 0.91)	0.25 (0.04 - 0.64)	0.72 (0.52 - 0.86)
Presence of fluid around the ovary	0.00 (0.00 - 0.34)	1.00 (0.84 - 1.00)	NA	0.73 (0.56 - 0.86)
Distended fallopian tube	0.00 (0.00 - 0.34)	1.00 (0.84 - 1.00)	NA	0.73 (0.56 - 0.86)
Sites of bleeding within the affected ovary	0.00 (0.00 - 0.34)	1.00 (0.84 - 1.00)	NA	0.73 (0.56 - 0.86)

**Table 5:** Frequency of the number of sonographic markers observed.

Number of markers	Frequency (N=37)	Percent	Cumulative Percent
0	2	5.4	5.4
1	11	29.7	35.1
2	9	24.3	59.5
3	10	27.0	86.5
4	4	10.8	97.3
5	1	2.7	100.0

**Table 6:** Sensitivity, specificity, and Youden score for combined markers.

Number of markers	Sensitivity	Specificity	Youden
1	0.9	0.44	0.34
2	0.7	0.7	0.4
3	0.4	0.96	0.36
4	0.1	1	0.1

## Data Availability

The data used to support the findings of this study are available from the corresponding author upon request.
